# Genomes from historical *Drosophila melanogaster* specimens illuminate adaptive and demographic changes across more than 200 years of evolution

**DOI:** 10.1371/journal.pbio.3002333

**Published:** 2023-10-12

**Authors:** Max Shpak, Hamid R. Ghanavi, Jeremy D. Lange, John E. Pool, Marcus C. Stensmyr

**Affiliations:** 1 Laboratory of Genetics, University of Wisconsin–Madison, Madison, Wisconsin, United States of America; 2 Department of Biology, Lund University, Lund, Scania, Sweden; 3 Max Planck Center on Next Generation Insect Chemical Ecology, Lund, Sweden; Fred Hutchinson Cancer Research Center, UNITED STATES

## Abstract

The ability to perform genomic sequencing on long-dead organisms is opening new frontiers in evolutionary research. These opportunities are especially notable in the case of museum collections, from which countless documented specimens may now be suitable for genomic analysis—if data of sufficient quality can be obtained. Here, we report 25 newly sequenced genomes from museum specimens of the model organism *Drosophila melanogaster*, including the oldest extant specimens of this species. By comparing historical samples ranging from the early 1800s to 1933 against modern-day genomes, we document evolution across thousands of generations, including time periods that encompass the species’ initial occupation of northern Europe and an era of rapidly increasing human activity. We also find that the Lund, Sweden population underwent local genetic differentiation during the early 1800s to 1933 interval (potentially due to drift in a small population) but then became more similar to other European populations thereafter (potentially due to increased migration). Within each century-scale time period, our temporal sampling allows us to document compelling candidates for recent natural selection. In some cases, we gain insights regarding previously implicated selection candidates, such as *ChKov1*, for which our inferred timing of selection favors the hypothesis of antiviral resistance over insecticide resistance. Other candidates are novel, such as the circadian-related gene *Ahcy*, which yields a selection signal that rivals that of the DDT resistance gene *Cyp6g1*. These insights deepen our understanding of recent evolution in a model system, and highlight the potential of future museomic studies.

## Abstract 2

Genomes from 25 museum specimens of Drosophila melanogaster (some more than 200 years old) reveal that small populations occupying northern Europe gave way to well-connected fly populations across the continent, and also illuminate targets of recent selection, including genes that may have helped this species adapt to novel climates, viruses, and insecticides.

## Introduction

Museum collections around the world contain billions of specimens collected over the last centuries. These collections serve as a window back in time—to an era before industrialization and modern agricultural practices—and could as such provide insights into issues ranging from insecticide resistance to climate change. The analysis of so-called historical DNA (hDNA), including from museum samples, accordingly holds much promise, but obtaining high-quality genomic data from older specimens remains a significant challenge [1,2].

A handful of prior studies have targeted whole-genome sequences from museum insect specimens. These have included proof-of-concept sequencing studies [3,4] and investigations of the taxonomic status of museum specimens [5–7], with 1 study including butterfly specimens up to 150 years old [8]. A few insect museomic studies have sequenced multiple individuals to examine potential targets of recent positive selection, such as focusing on the responses of honey bees to the introduction of a parasitic mite [9,10] and the emergence of the Colorado potato beetle as a crop pest [11].

*Drosophila melanogaster* is a primary model system for population genetics. Recently, the availability of data from multiple time points has begun to improve the prospects for distinguishing natural selection from neutral evolution in this system. Genome sequencing of wild-caught and laboratory-maintained *D*. *melanogaster* individuals has enabled the study of genomic evolution across seasonal (e.g., [12,13]) and decadal [14] time scales. However, no previous study has investigated genome-scale variation from historical *D*. *melanogaster* specimens nor from any species of such a minute animal.

**Fig 1:**
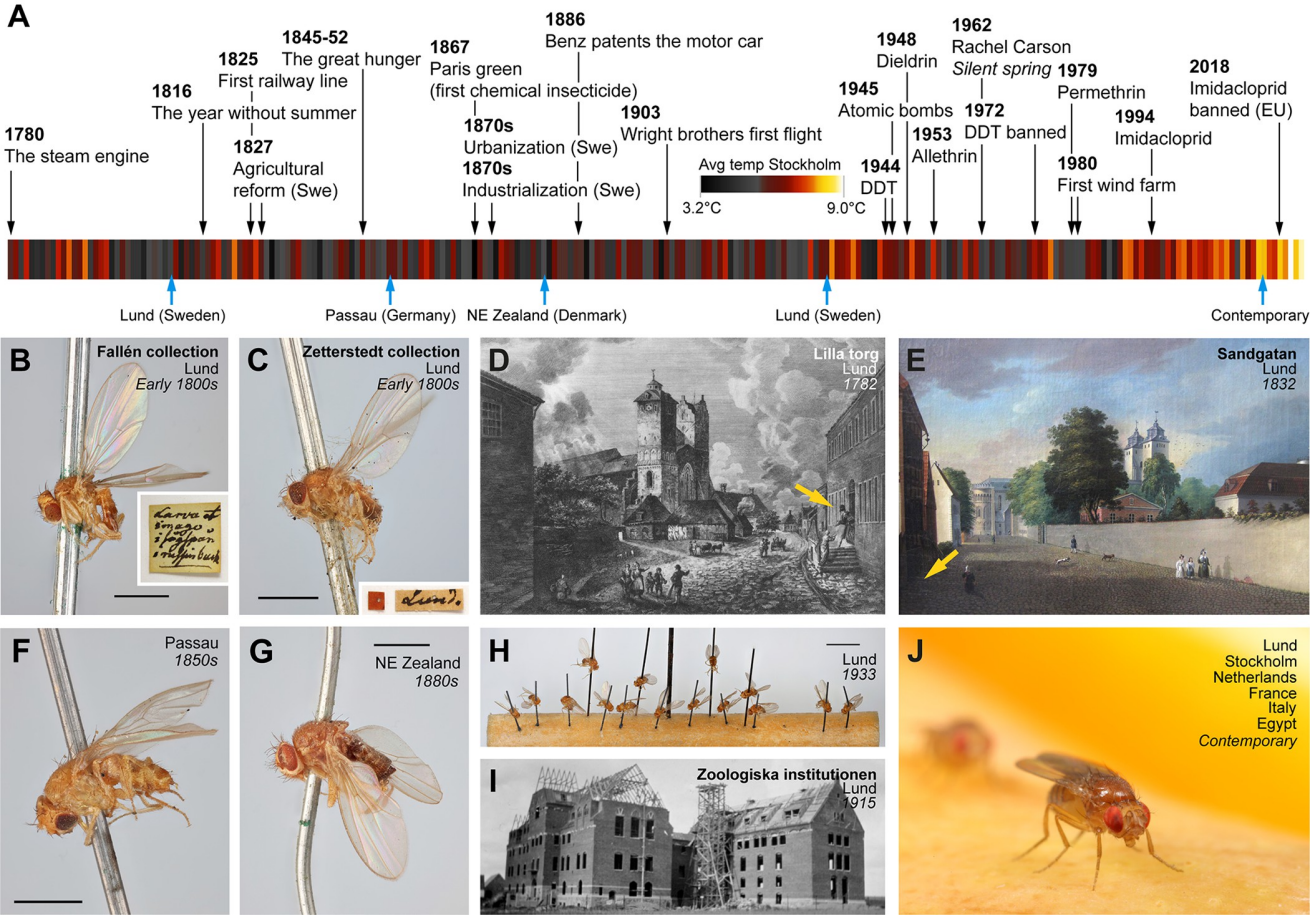
Historical context of the museum specimens and time interval encompassed by the present study. (A) Timeline showing the chronology of the analyzed fly samples, notable human events, and climate trends in Sweden (temperature data from SMHI, Sweden). (B) One of the 4 *D*. *melanogaster* specimens from the Fallén collection at the Swedish Natural History Museum (Stockholm). Insert shows the label (in Fallén’s handwriting) accompanying the specimen. Scale bar: 1 mm. (C) One of 3 *D*. *melanogaster* specimens from the Zetterstedt collection at the Biological Museum (Lund). Insert depicts the attached label. Scale bar: 1 mm. (D) Lydbecksa Gården in Lund, where Fallén had his lodgnings until 1811. (E) Sandgatan 5 in Lund, where Zetterstedt lived. (F) *D*. *melanogaster* specimen from the Natural History Museum of Denmark (Copenhagen), collected by Joseph Waltl in Passau, Germany. Scale bar: 1 mm. (G) *D*. *melanogaster* specimen from the Natural History Museum of Denmark (Copenhagen), collected by William Schlick on NE Själland, Denmark. Scale bar: 1 mm. (H) Fifteen *D*. *melanogaster* specimens from Lund, kept in collections of the Biological Museum (Lund). Scale bar: 5 mm. (I) The old department of Zoology building at Lund University. (J) A representation of the 6 contemporary population samples included in the present analysis. Photos: (B, C, F–H) C. Fägerström, (D) Leonard Axel Jägerskiöld/Göteborgs Naturhistoriska Museum, (E) Joseph Magnus Stäck/Lund University, (I) Lund University, (J) M. Stensmyr.

Here, we obtain and analyze genomes from historical *D*. *melanogaster* specimens of approximately 100 to 200 years in age, using minimally destructive techniques. The roughly 200 year time frame of our analysis should encompass the earliest stages of this ancestrally tropical species’ adaptation to a novel high latitude environment [15], along with profound human-mediated changes to the environment of this commensal species (**Fig 1A**). Hence, museomic study of European *D*. *melanogaster* offers the potential to reveal both demographic and adaptive changes during this dynamic time interval, against the backdrop of a well-annotated genome with detailed knowledge of gene function.

The insect collections held in Lund and Stockholm are among the oldest in the world and contain many important samples, including the specimens used to erect the genus *Drosophila* (Fallén, 1814 to 1826) and multiple early 1800s specimens of *D*. *melanogaster*. These include 4 specimens from the collection of Carl Fredrik Fallén of Lund University (1764 to 1830; **Fig 1B**). From the same time period, 3 specimens exist from the collection of Johan Wilhelm Zetterstedt (1785 to 1874, **Fig 1C**), a student of Fallén, and from 1822 professor at Lund University. This material comprises the lectotype (not examined here) and 2 paralectotypes of *Drosophila approximata*, a junior synonym of *D*. *melanogaster*.

The precise timing, location, and collector for the 6 examined specimens is not easy to decipher. The Fallén samples, collected in Lund, are accompanied by an original note, stating (translated) “*Imago and larvae in sawdust [*unintelligible*] can of raisins*.” If the flies were caught by Fallén himself, a potential locality would then be his lodgings in central Lund (**Fig 1D**), and that the material was collected before 1811, when Fallén began to spend considerable time elsewhere. His first mention of *Drosophila* can be found in a letter to Zetterstedt dated 14 September 1810 (Zetterstedt archive, Lund University). The paralectotype samples examined from Zetterstedt’s collection are labeled Lund and Smol (i.e., Småland) respectively, and they are likewise difficult to precisely date. Based on Zetterstedt’s *Diptera Scandinaviae disposita et descripta*, he collected *D*. *approximata* in Lund (possibly at his home in central Lund; **Fig 1E**). The specimen examined here from Småland (and likewise the lectotype) was attributed to Sven Ingemar Ljungh (1757 to 1828), civil servant and prominent naturalist, who resided at Skärsjö manor (Jönköping County, Småland—roughly 200 km from Lund). It is unclear whether these specimens are the ones mentioned in his treatise on Scandinavian Diptera, but if so, the flies would likely have been caught within a decade or so of Fallén’s samples. However, the owner of a given specimen may or may not have been its collector (e.g., Zetterstedt kept many of Fallén’s specimens in his collection). These Swedish specimens are, to the best of our knowledge, the earliest *D*. *melanogaster* available in any collection, potentially predating the Meigen holotypes kept in Paris by 2 decades.

From the collection in Copenhagen, there are 2 specimens from Passau, Germany (**Fig 1F**) collected by Joseph Waltl (1805 to 1888), undated but likely collected in the 1850s. Also from Copenhagen, there is 1 specimen collected by Rasmus William Traugott Schlick (1839 to 1916) on northeast Zealand, Denmark (**Fig 1G**), undated but likely collected in the 1880s. Lastly, we have a set of 15 specimens from the collection in Lund (**Fig 1H**), collector unknown, labeled “Zootis 1933”—the informal name of the former Zoological Department at Lund University (**Fig 1I**).

In all, the Swedish and Danish museum collections contain 26 *D*. *melanogaster* specimens suitable for analysis. We here provide whole-genome analysis from these flies, obtaining from most of them relatively complete and high-quality genomes, and we compare these against modern fly genomes (**Fig 1J**). The roughly 200 year time frame of this analysis may encompass 3,000 fly generations [16,17], analogous to studying human evolution across 75,000 years (approximately the time before present when modern humans first colonized Eurasia from Africa (e.g., [18–21])). Examining changes in genomic diversity across 2 roughly century-scale time intervals, we find that the relationship between the Lund population and other European populations has changed over time. We identify a variety of strong candidates for the action of positive selection in each time interval, providing temporal context for previously known cases of selection, while also identifying novel putative selection targets. These findings illustrate the potential of museomic studies to deepen our understanding of recent evolution in a rapidly changing world.

## Results and discussion

### Most historical flies yielded genomes of comparable quality to contemporary flies

**Fig 2:**
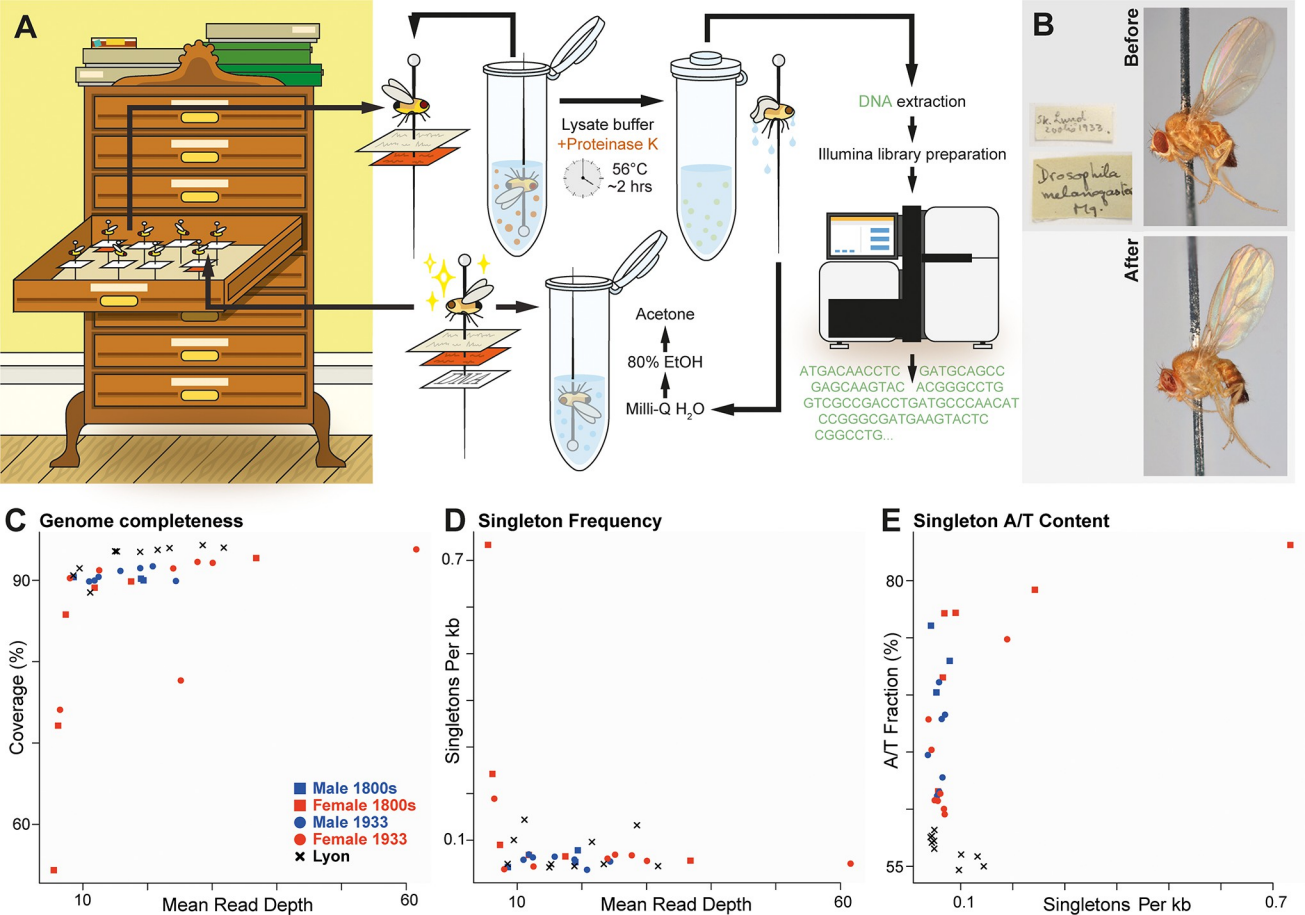
Sequencing of historical *Drosophila* specimens yields genomes with slightly reduced genomic coverage and some evidence of cytosine deamination. (A) Illustration of the process of extracting and sequencing DNA from pinned insect specimens. Flies were submerged in a lysis buffer for 2 h, then put through 3 washing steps and dried, before being restored to the museum repository. Genomic libraries were then prepared and sequenced using the Illumina NovaSeq 6000 platform. Further details appear in the Methods section. (B) Images of the appearance of a representative specimen (from 1933) before and after the DNA extraction protocol, illustrating minor changes in coloration. Photo: C. Fägerström. (C) The fraction of genomic coverage is plotted as a function of mean per-site read depth for each historical fly genome and for a representative set of contemporary inbred line genomes from Lyon, France. For a given level of depth, most historical fly genomes achieve levels of genomic coverage only slightly lower than contemporary genomes. (D) The relationship between depth and unique (singleton) variant-calling. Only the lowest quality historical fly genomes show notable elevations in the rate of singleton variants called. (E) The fraction of singleton variants called as adenine or thymine is elevated for historical fly genomes, particularly those with greater singleton rates, potentially due to cytosine deamination. Data underlying panels C–E can be found in S1 Table.

For proof-of-principle, we first attempted to extract DNA from one of the 1933 “Zootis” specimens (**Fig 1H**). Briefly, the whole fly—still attached to the pin—was first incubated in a lysis buffer at 56°C for 2 h, after which the fly was washed, dehydrated, and returned to the collection (**Fig 2A**). The extraction process left the fly largely unharmed, except for a slight loss in coloration and some shrinkage of the abdomen (**Fig 2B**). The extracted DNA was subsequently prepared for Illumina short read sequencing. As expected, the DNA was highly fragmented with an average fragment length of about 50 bp. The Illumina run yielded approximately 24 million reads, and after reference mapping, the ensuing genome was 91% complete with a 15.7× mean sequencing depth. In short, the employed method was indeed able to extract enough DNA to piece together a largely complete genome, while doing minimal harm to the specimen.

Having verified the method, we applied it to the other 25 specimens, and we were able to generate genomes from all but one. While the mean depth of coverage per site varies among the historical fly genomes (**Fig 2C**), the median value among these genomes was approximately 20×, which is comparable to typical *D*. *melanogaster* genomes generated from contemporary source material [22]. We found, however, that with similar mean depth, genomic coverage tends to be slightly lower in historical versus contemporary genomes (often with only 2% to 3% less of the genome covered; **Fig 2C** and **S1 Table**).

The modest reduction in genomic coverage for most of the historical fly genomes may relate to their consistently shorter insert sizes—typically only about 50 bp (**S1 Table**), compared to contemporary genomes that are generally sequenced from fragments of a few hundred bp in length. The shorter DNA fragments from the old specimens are expected to limit the potential of reads to map uniquely in repetitive genomic regions, and hence such regions may not receive base calls in the historical genomes, leading to somewhat reduced genomic coverage as observed above. Genomes from the 1800s flies had mean insert sizes averaging 47.6 bp, while those from the 1933 collection averaged 50.8 bp. Although the distributions of mean insert sizes overlap between time points, they nevertheless differ significantly (*P* = 0.0188; Mann–Whitney two-tailed *U* test). There was a marginal correlation between fragment length and depth of coverage (correlation *r* = 0.40, *P* = 0.055). A stronger and significant association was found between fragment length and genomic coverage (*r* = 0.63, *P* = 0.0001).

The shorter length of historical fly DNA fragments may be due to DNA degradation, which may also explain the reduced depth and genomic coverage obtained from a minority of the examined specimens. Among the nine 1800s samples, 4 genomes were characterized by a low average read depth of under 10 reads per site and/or a low genomic coverage of less than 80%. From the 1933 collection, 1 additional sample was excluded from further analyses due to extremely low coverage and read depth. Most samples with low coverage also had low mean read depth (**Fig 2C** and **S1 Table**), and the overall correlation between these quality metrics was statistically significant (*r* = 0.404, *P* = 0.018). We note that all 7 low-quality genomes (as defined above) were from female flies (out of 14 total females), whereas all 11 males yielded genomes with higher coverage and depth. This sex difference is statistically significant (two-tailed binomial *P* = 0.0057) and may be due to female flies being larger and potentially experiencing greater decay before dehydration.

Regarding the 6 historical fly genomes retained with lower sequencing depth, inferences of diploid genotype are not statistically robust at sites for which an individual has few reads. Therefore, we treated the lower quality genomes as though they were haploid for autosomal and female X chromosomes by assigning only a single, most frequent nucleotide at each site. Consequently, there were effectively 14 rather than 18 autosome alleles for the 1800s samples and 28 rather than 30 for the 1933 autosomes. Similarly, treating each low-quality female X as haploid reduced allele counts to 11 and 21 for 1800s and 1933 X chromosomes, respectively (from what would otherwise be X chromosome allele counts of 15 and 23).

The DNA degradation inferred above could also lead to incorrect base identification via anomalous sample-specific variants at individual sites. Therefore, we assessed whether singleton variants (defined as an allele called only once among the full panel of 1800s, 1933, and contemporary genomes analyzed, for the subset of sites with no missing data among these genomes) were enriched in samples with low depth and coverage. Indeed, we found a higher relative incidence of singleton variants unique to genomes with lower genomic coverage (two-tailed binomial *P* < 0.01; Fig 2B) and to a lesser extent those with low depth as well (*P* = 0.05).

One of the most common types of nucleotide degradation is cytosine deamination, which can lead to spurious thymine enrichment, which has been documented extensively in ancient DNA samples (e.g., [23,24]). We therefore assessed the extent to which A/T nucleotides are overrepresented among singletons from each genome. We found a higher fraction of A/T sites in singletons among the historical samples than in modern genomes (**Fig 2D and 2E and** **S1 Table**), which could reflect the presence of some degradation-associated errors. Whereas the modern genomes had singleton A/T proportions of approximately 55%, many of the historical genomes’ singletons were 60% to 74% AT, presumably due to cytosine deamination. Although most historical genomes do not show meaningfully elevated singleton rates, our findings regarding singleton AT% justify the exclusion of singleton alleles for analyses that are sensitive to rare alleles and small frequency differences, such as demographic inferences based on allele frequency change over time. In contrast, analyses that search for window-scale outliers for elevated allele frequency differentiation between samples should be little affected by the low rates of spurious singleton variants indicated by these analyses.

### The historical fly genomes show signs of relatedness and inbreeding

**Fig 3:**
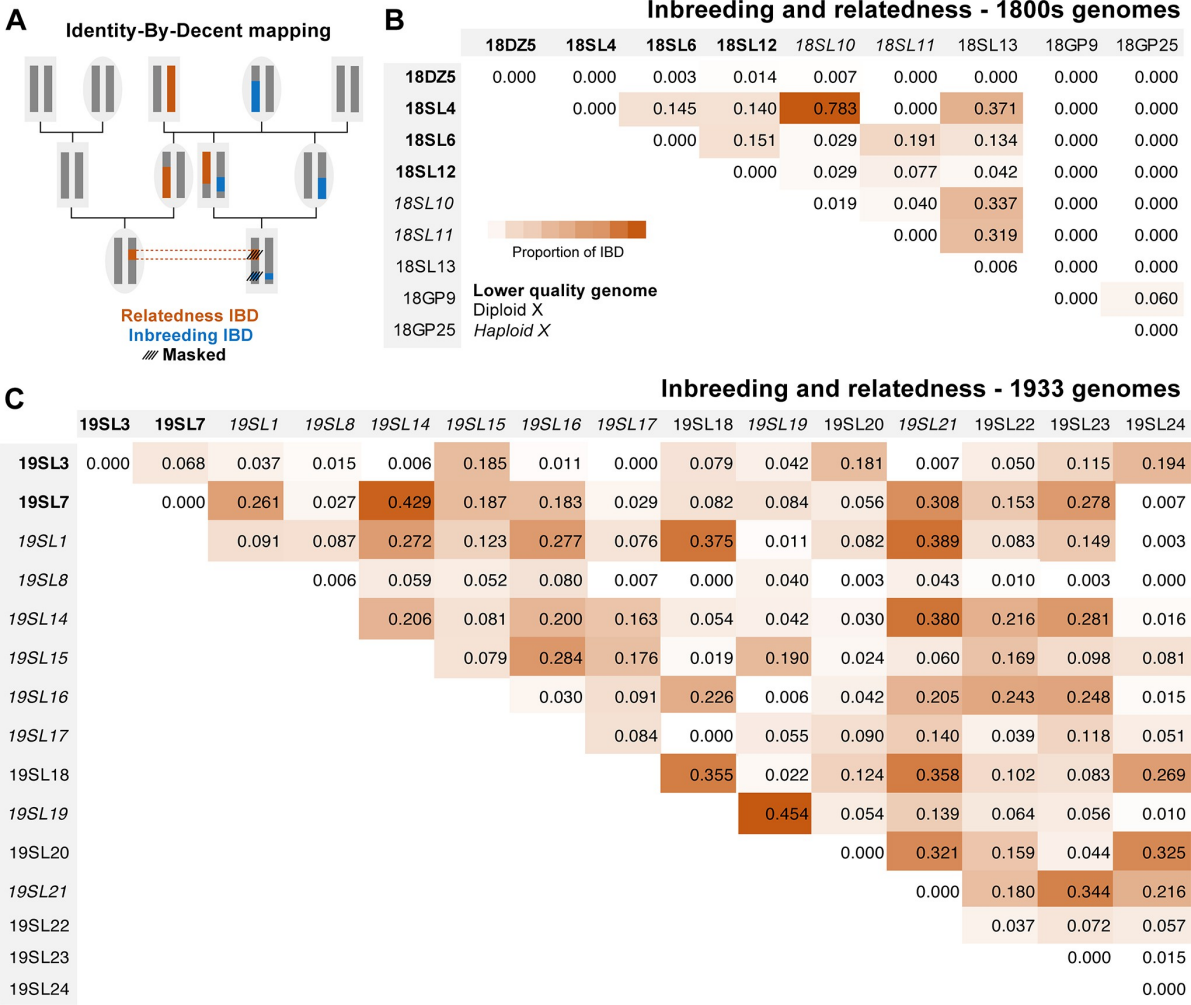
Significant levels of IBD were observed within and between many historical fly genomes, reflecting inbreeding and relatedness. (A) Illustration of the genealogical relationships that may result in IBD due to relatedness between individuals (brown) or inbreeding within individuals (blue). Dashed lines illustrate masked segments, excluded from downstream analysis. (B) For the 1800s Sweden-Lund and Germany-Passau samples, the proportions of each genome detected as IBD due to apparent inbreeding (given along the diagonal) were low or zero. Above the diagonal, the proportions of each pairwise genomic comparison detected as IBD due to apparent relatedness varied within the Lund sample from zero to over 75% (the latter indicating a close familial relationship), while the 2 Passau genomes showed low-level relatedness IBD. Individuals are labeled based on their collection century, location, and arbitrary individual number—e.g., 18SL4 is 1800s Sweden-Lund individual 4, whereas DZ and GP refer to the Denmark (Zealand) and Germany (Passau) samples, respectively (**S1 Table**). (C) The same quantities are given for the Lund 1933 population sample, revealing some genomes with high levels of inbreeding, and a broad spectrum of relatedness among genomes. Since the probability of IBD detection is different for diploid and haploid sequences, males (with a single X chromosome) are given in italics. Lower quality genomes treated as haploids are shown in bold. IBD regions were masked in subsequent analyses.

A preliminary analysis of pairwise genetic distances among the genomes indicated that some specific pairs of individuals from within the early 1800s and 1933 Sweden samples had genetic similarity implying relatedness (**S2 Table**). Because the inclusion of related individuals in estimates of allele frequency generates artifactual nonindependent sampling, we sought to identify and mask individual chromosomal intervals displaying “identity by descent” (IBD) from downstream population genetic analyses (**Fig 3A**).

We identified IBD regions between a given pair of individuals based on windows in which they had unusually low genetic distance between them (closer to the distance expected if at least 1 haplotype from each individual was IBD than to the distance expected for unrelated haplotypes). We inferred IBD windows spanning various lengths, up to whole chromosome arms (**S2 and S3 Tables**). Results indicated varying levels of relatedness among individuals within the 1800s and 1933 Sweden samples, up to levels expected for first or second order relatives, and a much lower level of relatedness between the 2 specimens from 1840s Germany (**Fig 3B and 3C**). In addition to IBD between genomes, we found that a few of the 1933 Lund samples had higher levels of within-genome IBD due to inbreeding—several samples from 1933 had long regions of chromosomes with depleted heterozygosity (**Fig 3C and S2 and S4 Tables**), consistent with mating between close relatives.

The 6 Swedish flies from the early 1800s also all showed relatedness, with some pairs even at sibling-level IBD. Since close relatedness is only expected from spatially and temporally proximate samples, we conclude that in spite of museum records tentatively linking them with 3 different scientists and 2 localities, these specimens were all from the same Lund collection event. These results may reflect sharing of specimens among contemporary scientists and/or imperfect record keeping through time.

The extensive IBD observed here contrasts with the low levels of relatedness observed from contemporary population samples, even when multiple fly lines founded from the same trap site were analyzed [22]. The elevated IBD of the historical fly genomes could have resulted from sampling methodology (e.g., propagation of collected insects in the lab) and/or from a lower population density of flies in earlier eras (as suggested below).

We masked individual chromosomal intervals to prevent IBD from biasing downstream analyses—masking one individual’s genotypes within each pairwise relatedness IBD block, and counting only 1 allele per individual within an inbreeding IBD block (see Methods). Following IBD masking, we were left with averages of 9 to 11 sampled chromosomes for autosomal arms for the combined 1800s samples (versus 14 before masking) and 10 to 14 (versus 28) for the 1933 samples. The average sample size of X chromosome sites was then 8 for the 1800s set and 11 for 1933, versus 12 and 20 before masking. Based on the mosaic pattern of window masking due to intergenomic and intragenomic IBD across individuals on each chromosome arm, post-filtering sample size varied around these averages, and downstream analyses included sample size thresholds for site inclusion (see Methods).

### Genomic diversity suggests transiently elevated genetic differentiation

**Fig 4:**
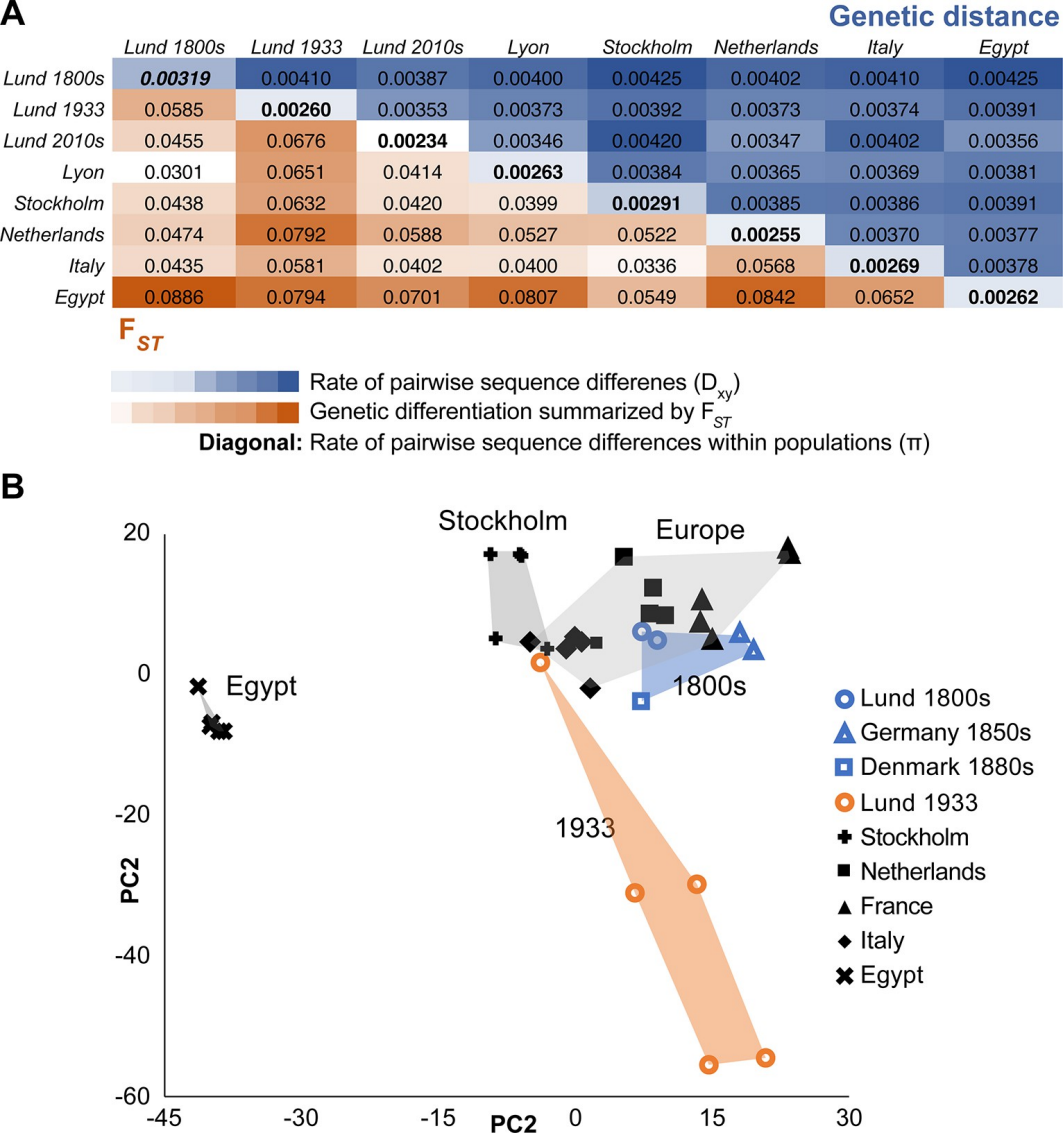
Genetic differences among historical and modern fly populations suggest transient differentiation in the Lund population. (A) Genetic differentiation between population samples is summarized by *F*_*ST*_ (below diagonal, red heat map) and by the rate of pairwise sequence differences *D*_*xy*_ (above diagonal, blue heat map). Along the diagonal, the rate of pairwise sequence differences within populations (nucleotide diversity) is given. Data reflects the X chromosome only (to avoid effects of inversions), with down-sampling to 4 alleles per population at each site. The Lund population appears to show decreasing diversity with time, subject to caveats regarding historical genome quality and the differentially processed Lund 2010s pool-seq data. When Lund allele frequencies are compared to other European populations (via *F*_*ST*_), differentiation increases between the 19th and 20th century Lund samples, then decreases again between the 20th and 21st century samples. (B) PCA results are shown for the same individually sequenced population samples included in (A), in terms of loadings from the first 2 principal components. Here again, the Lund 1933 population shows elevated differentiation, whereas genomes from the three 1800s locations cluster with modern European genomes. These results reflect an analysis of X chromosome variation, with each population sample downsampled to 5 individuals, with non-inbred historical genomes lacking close relatedness chosen for this analysis. Data underlying this figure panel can be found in S5 Table.

We then sought to examine how within-population genetic diversity and between-population genetic differentiation have changed across more than 2 centuries. We focused initially on the X chromosome in order to avoid the influence of inversions (e.g., [25]). Patterns of nucleotide diversity (*π*) in Lund appeared to show a decline with time (**Fig 4A**), which could reflect the action of genetic drift. However, we note that damage-induced errors may inflate diversity estimates, especially for the 1800s sample (as potentially indicated by its slightly elevated D_xy_ values). In addition, the modern Lund sample represents a published pool-seq data set, analyzed via a distinct pipeline including the masking of rare variants, which may deflate its diversity estimate.

Patterns of genetic differentiation (as indicated by *F*_*ST*_) revealed a curious temporal trajectory (**Fig 4A**). When Lund samples are compared to modern samples from around Europe, between the 1800s and 1900s sampling points, Lund became more differentiated from other populations. In contrast, between the 1900s and 2000s sampling points, Lund became more similar to other modern European populations. Concordant patterns were observed from principal components analysis (PCA) based on X-linked variation (**Fig 4B** and S5 **Table**). Here, 1800s Lund genomes were seen to cluster with modern European genomes, whereas the 1933 Lund genomes form a distinct cluster. This unexpected pattern of transient local differentiation could indicate that distinct evolutionary forces had predominant influences on genomic diversity between these time points.

Increased genetic differentiation during the 1800s to 1933 period could reflect an elevated influence of genetic drift. This interval likely represents the earliest days of *D*. *melanogaster* occupying northern Europe [15], perhaps as shifting human activities only just permitted the species to survive in this region, and hence we might predict that the initial abundance of this species was low. The climate during this period was also colder than at present (**Fig 1A**), which may have also limited local population sizes. Consistent with this hypothesis, Zetterstedt [26] described the species as rare in southern and middle Sweden, and it was absent from the earlier Scandinavian insect descriptions of Linnaeus [27,28] and Fabricius [29].

In contrast, the genomic homogenization between Lund and other European regions that occurred between 1933 and the 2010s could reflect increased migration through time, in conjunction with reduced genetic drift in fly populations that may have grown with time. Both of those demographic changes might be predicted based on concurrent changes in human activity during the 20th century. This time period featured a 5-fold increase in the human population of Lund and the surrounding region, along with a warming climate (**Fig 1A**), both of which may have been conducive to population growth in this human commensal insect (and hence reduced drift). In parallel, increased human transportation, particularly the expanded shipping of fruit and other commodities, would be expected to facilitate increased long-distance dispersal of *D*. *melanogaster*. We note that such temporal shifts in demography would not be detected by conventional demographic inference based on modern samples, and it is unclear how well simple demographic models can recapitulate the effects of more complex histories on genomic diversity.

We also examined whether changes in the frequencies of common polymorphic inversions may have influenced genetic diversity between time points (see Methods). We found only a single copy of *(2L)t* from one 1933 Lund genome, and no inversions among the 1800s genomes (**S6 Table**). In the modern Lund sample, *(2L)t* is at 13% frequency, while other tested inversions appear to be absent (**S6 Table**). Hence, although we cannot rule out changes in inversion frequencies through time, inversions do not appear to be a likely driver of genomic differentiation between time points.

We further investigated a simple model of the demographic history of Lund by using a Bayesian approach to identify the best-fitting effective population size (*N*_*e*_) for each time interval. For each chromosome arm separately, we used simulations to identify the value of *N*_*e*_ that best matched the empirical mean change in allele frequency at non-singleton SNPs between time points (see Methods). In contrast to longer-term estimates of *N*_*e*_ in this species, which are often on the order of 1 million (e.g. [30]), our point estimates of Lund *N*_*e*_ were only 2,500 to 3,300 diploid individuals for the 1800s to 1933 time interval and 2,400 to 3,200 for the 1933 to 2010s interval (**S7 Table**). These values are of the same order of magnitude as an estimate of 9,500 from a northeast United States population between 1975 and 2015 [14]. Estimates from both studies share, however, a key limitation in assuming that genetic drift (along with random sampling variance) is responsible for all observed frequency differences. To address how well this assumption holds, we compared *π* from coalescent simulations based on our estimated *N*_*e*_ values to those from the empirical data, using a demographic model previously estimated for a French population [30] as a starting point for the pre-1800s history. While this history allowed us to match the Lund 1800s *π* reasonably well, our low estimates of *N*_*e*_ yielded simulated values of *π* that were less than half of those actually observed in our empirical data (**S7 Table**). These results indicated that drift-only models did not provide an accurate approximation of the Lund population’s demographic history and that true *N*_*e*_ values were probably greater than our estimates. Instead, migration may have played an important role in shifting allele frequencies without decreasing *π*, as suggested above. Estimating a reasonable spatiotemporal demographic model that incorporates both local population sizes and geographic patterns of genetic structure and gene flow may require more extensive sampling of population genomic data across space and time.

### Genome scans to detect recent shifts in allele frequencies

**Fig 5:**
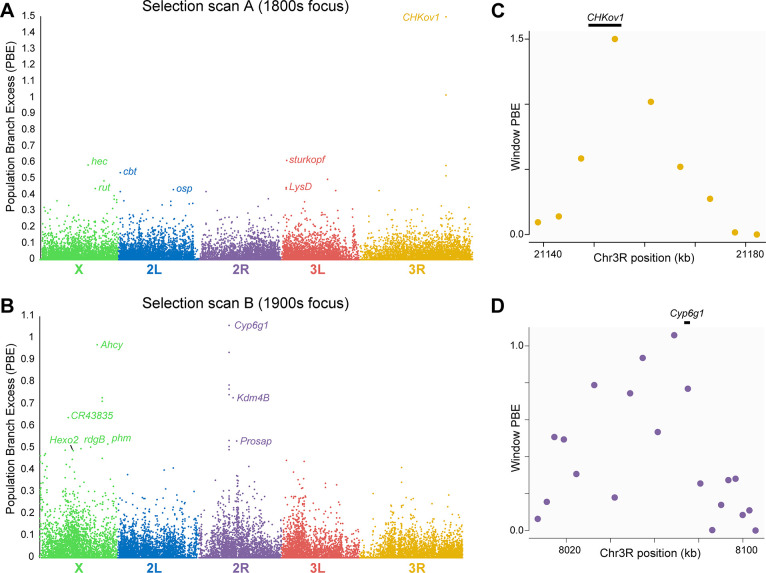
Allele frequency changes across centuries reveal candidates for positive selection. (A) Allele frequency change for approximately 5 kb genomic windows, focusing on the 1800s–1933 time interval (scan A), as quantified by PBE. Genes within top outlier regions that are discussed in the text are indicated. (B) PBE is shown for the same genomic windows, focused on the 1933–2010s time interval (scan B). (C) Window-level PBE is shown for a roughly 50 kb region focusing on the top outlier region from scan A, with the position of the known selection target *ChKov1* indicated. (D) Window-level PBE is shown for a roughly 100 kb region focusing on the top outlier region from scan B, with the position of the known selection target *Cyp6g1* indicated. Data underlying this figure can be found in **S8 Table**.

One principal goal of this study is to identify instances of elevated genetic differentiation between time points that may reflect the action of recent positive selection. Our SNP-based genome scan focused on population branch excess (PBE [31]), a statistic that quantifies elevated allele frequency differentiation in a focal population compared to 2 other reference/outgroup populations. PBE builds upon the *F*_*ST*_-based population branch statistic (PBS [32]) but is more tailored to detecting selection that is specific to the focal population. To search for selection between the early 1800s and 1933, we defined the 1800s samples as the focal population and included the Lund 1933 and Lund 2010s samples as outgroups. To focus on the 1933 to 2010s interval, Lund 1933 was the focal population and Lund 2010s and France were the outgroups. PBE was evaluated in diversity-scaled windows averaging 4.6 kb in length. In the absence of a suitable demographic model to provide a null hypothesis for the extent of genetic differentiation, we focused our analysis on the top 1% of window PBE values. However, we emphasize that any outlier-based genome scan may entail both false positives and false negatives. Our SNP-based PBE scans revealed notable outlier peaks reflecting elevated allele frequency change for each time period (**Fig 5A** and **5B**). Overall, we identified 190 outlier regions for the 1800s to 1933 time interval (referred to below as scan A) and 173 for the 1933 to 2010s interval (scan B), with some of these regions incorporating multiple outlier windows (**S8 Table**). Since not all outliers may represent true positive targets of recent natural selection, we only discuss loci that had among the most extreme frequency changes across each century, referring to these outlier regions based on their ranked maximal window PBE values. This restricted focus is motivated by uncertainty regarding the actual number of loci under selection in each time interval, which we do not attempt to estimate due to limitations in the data and in our ability to identify a realistic neutral model.

### Top SNP-based outliers inform classic examples of positive selection

In both time intervals, the top window PBE value was also associated with the widest outlier region, which is consistent with relatively strong positive selection. For the 1800s to 1933 interval, the top outlier (labeled A1) was a 31 kb region that included the gene *CHKov1* (**Fig 5C**), which is thought to encode an ecdysteroid kinase [33]. This gene is known to be associated with a protein-truncating transposon insertion under recent positive selection in non-African *D*. *melanogaster* populations, which was reported to correlate with resistance to an organophosphate pesticide [34]. Transposon insertion and gene duplication at the *CHKov1*/*CHKov2* locus has, however, also been found to correlate with resistance to infection by the *D*. *melanogaster* sigmavirus (DmelSV) [35]. Given the lack of widespread insecticide usage during this period, the timing of selection indicated by our analysis rather favors the antiviral role of this locus as a potential selective advantage. It was estimated that *D*. *melanogaster* acquired this rhabdovirus around 200 years ago [36], which predicts that our 1800s interval should have included key stages in the evolution of resistance to DmelSV. Consistent with this hypothesis, we also found the gene *refractory to Sigma P* (*ref(2)p*) within outlier region A20. Ref2p activates the Toll pathway and has been shown to confer resistance to DmelSV [37].

*CHKov1* and *ref(2)p* are of particular interest because mutational variants associated with viral resistance have been previously identified [35,38]. In *CHKov1*, several SNPs have alleles in linkage disequilibrium with a transposon insertion in the gene [34] associated with viral resistance [35]. Based on 7 such SNPs scored in our data, we estimated that the resistant haplotype increased from 16.7% in the 1800s sample to 100% in the 1933 and 2015 Lund samples. The occurrence of 3 resistance-associated haplotypes among the 1800s genomes (carried in heterozygous form by 2 early 1800s Lund flies, as well as the later Zealand sample) could indicate that selection on this allele began prior to the collection of our 1800s flies. The fixation of the resistant haplotype in the 1933 and 2010s Lund samples suggests a complete (or nearly complete) sweep, mirroring findings from some, but not all recently collected *D*. *melanogaster* population samples [34].

The outlier PBE score for *ref(2)p* for the 1800s time interval appeared not to be driven by the short, complex deletion identified by [38]. Based on inspecting reads, this variant appeared to exist in 2 early 1800s Lund flies and two 1933 Lund flies, implying modest frequency change between temporal samples. The highest SNP-level PBE score at this gene was at a non-synonymous variant over 1 kb downstream from the complex deletion (at R5 position 2L:19544138, a threonine/serine polymorphism).

The top outlier for the 1933 to 2010s interval (denoted region B1) spanned 75 kb and included a known target of insecticide resistance evolution, *Cytochrome P450 6g1* (*Cyp6g1*, shown in **Fig 5D**). This gene is associated with resistance to dichlorodiphenyl-trichloroethane (DDT) and other insecticides in *D*. *melanogaster*. As with *ChKov1*, there is prior evidence for positive selection associated with transposon insertions and gene duplication [39–42]. Here, the novel and widespread deployment of DDT, introduced in 1944, provides a clear hypothesis for a selective pressure driving the observed frequency changes at SNPs linked to the locus in question.

### A subset of top outliers show narrow peaks of genetic differentiation

**Fig 6:**
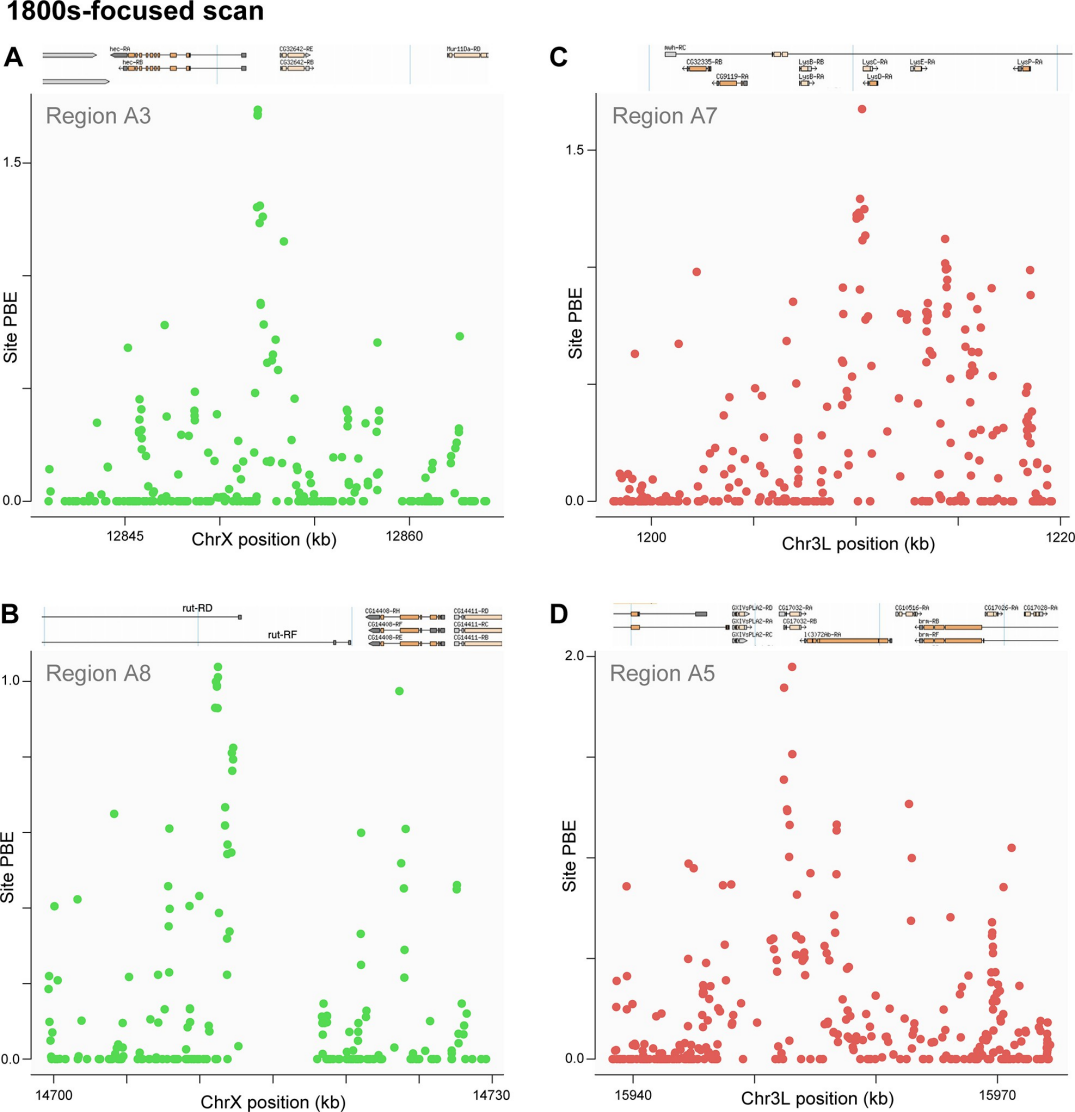
Narrow SNP-level intervals of differentiation indicate potential targets of natural selection from the 1800s-focused scan. PBE for each SNP is plotted across selected top outlier regions that showed narrow peaks of differentiation. These peaks occurred in or near: (A) *hector* (hec) in region A3, (B) *rutabaga* (rut) in region A8, (C) *Lysozyme C* (*LysC*) and *Lysozyme D* (*LysD*) in region A7, and (D) *CG17032* in region A5. Data underlying this figure can be found at: https://doi.org/10.5281/zenodo.8290185 .

**Fig 7:**
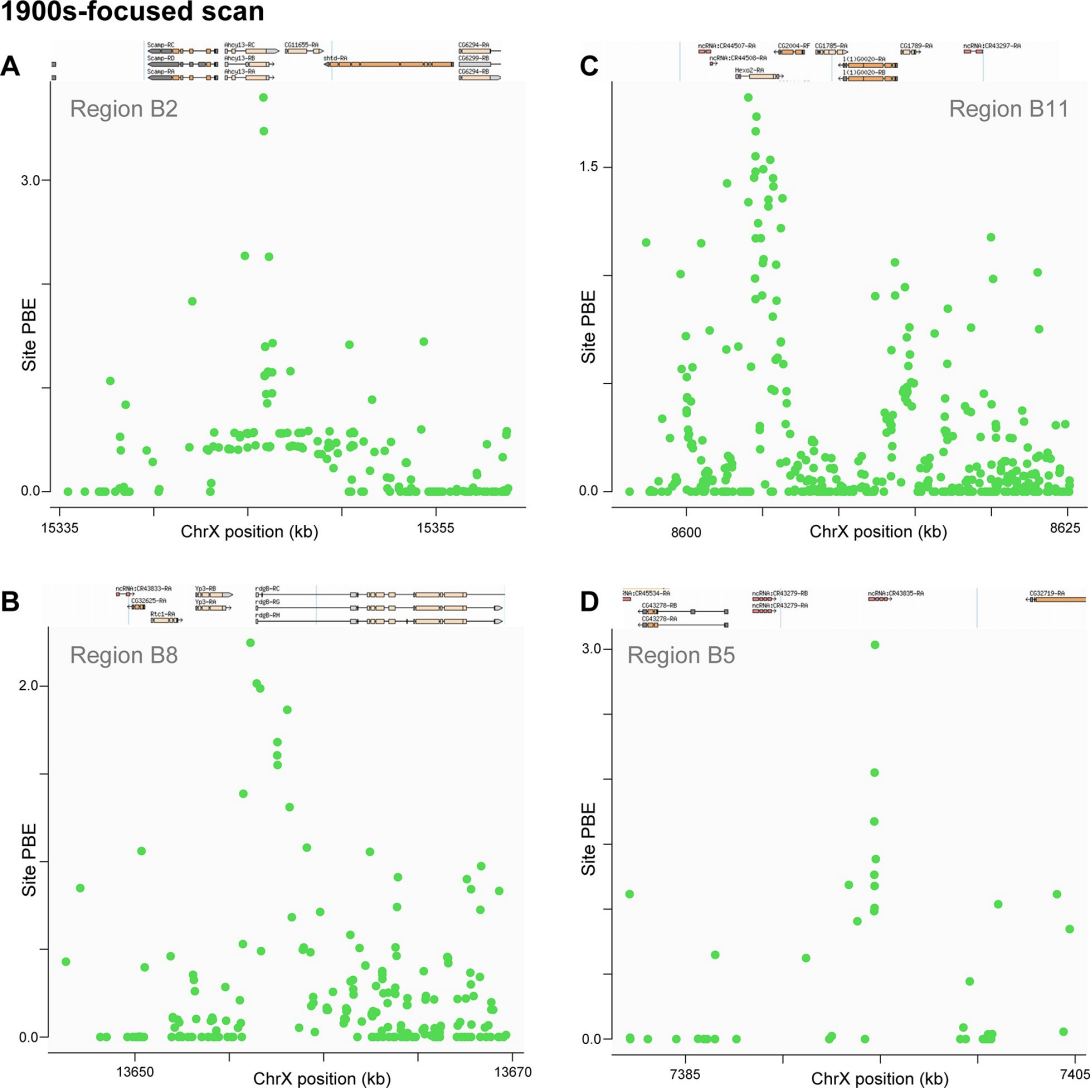
Narrow SNP-level intervals of differentiation indicate potential targets of natural selection from the 1900s-focused scan. PBE for each SNP is plotted across selected top outlier regions that showed narrow peaks of differentiation. These peaks occurred in or near: (A) *Adenosylhomocysteinase* (*Ahcy*, or *Ahcy13*) in region B2, (B) *retinal degeneration B* (*rdgB*) in region B8, (C) *Hexosaminidase 2* (*Hexo2*) in region B11, and (D) *ncRNA-CR43835* in region B5. Data underlying this figure can be found at: https://doi.org/10.5281/zenodo.8290185 .

Both of the above outlier regions were relatively broad, and some of the other top outliers also showed somewhat less-broad plateaus of elevated genetic differentiation between temporal samples. In contrast, several other top outliers showed more narrowly localized signals of elevated genetic differentiation. At least in spatial analyses of local adaptation, broader intervals of genetic differentiation are more likely to be associated with selection favoring a single initial haplotype (resulting in a hard sweep), whereas narrow signals of frequency change may result from selection favoring multiple initial haplotypes, resulting in a “soft sweep” [43]. Narrow differentiation signals are of particular interest based on their potential to indicate not just a specific gene but often a small set of candidate variants which may include the target of selection, and we therefore present the clearest cases among top outlier regions in which signals of maximal differentiation point to narrow regions. **Fig 6A–6D** depicts 4 narrow SNP-level patterns of allele frequency change focused on the 1800s to 1933 time interval, whereas **Fig 7A–7D** depicts 4 such examples for the 1933 to 2010s interval (all of which were among the top 11 regions for window PBE for their time interval). Focusing on the earlier period, a few SNPs upstream of *hector* (*hec*; in region A3), a calcitonin-like neuropeptide receptor involved in male courtship behavior [44], showed elevated PBE values (**Fig 6A**). In region A8, a cluster of SNPs within a 5′ UTR intron showed the highest PBE values at *rutabaga* (*rut*; **Fig 6B**), a calcium-responsive adenyl cyclase that influences learning, memory, lifespan, circadian rhythm, ethanol tolerance, and response to heat and oxidative stress [45–49]. We also detected a narrow set of SNPs within region A7 at the lysozyme genes *LysC* and *LysD* (**Fig 6C**), a locus for which a gene duplication and inversion polymorphisms have been reported [50], and which was found to be strongly differentiated between Swedish and Italian *D*. *melanogaster* populations [51]. In contrast, no function is known for *CG17032* (region A5; **Fig 6D**), for which the highest PBE value was at a synonymous variant (at R5 position 3L:15953092). Among top 1800s to 1933 outliers with more diffuse SNP-level signals, the A2 region encompassed *sturkopf*, a lipid droplet-associated protein that regulates growth and stress response [52] and is known to harbor a polymorphic duplication associated with low genetic diversity [53]. Region A4 included *cabut* (*cbt*), a transcription factor that regulates metabolic and circadian responses to nutrition [54].

For the 1933 to 2010s interval, a window focused on *Adenosylhomocysteinase* (*Ahcy*; in region B2) yielded a PBE value nearly as extreme as that observed at *Cyp6g1*, but with a far narrower genomic scale (**Fig 7A**). Curiously, the top SNP PBE value occurs at a synonymous variant (at R5 position 3L:15953092). *Ahcy* regulates methionine metabolism, histone methylation, and lifespan [55]. It is preferentially expressed in circadian-regulating pacemaker neurons [56], and its role in circadian regulation has been confirmed in mice [57]. Despite the strength of this signal, *Ahcy* does not appear to have been detected as a top selection candidate by previous studies. The next-highest region (B3, not plotted), *Lysine demethylase 4B* (*Kdm4B*) also impacts histone methylation and circadian rhythm [58]. Beyond this pair of loci, *Prosap* from this same 1933 to 2010s time interval (B6), along with *cbt* (A4) and *rut* (A8) from the 1800s to 1933 scan, also represent top outliers with circadian functions. The evolution of circadian behavior in high latitude *D*. *melanogaster* populations is well known (e.g., [59,60]). The northerly location of Sweden entails pronounced seasonal variation in day length, a cue that helps regulate reproductive diapause [61], which is a key adaptation in *D*. *melanogaster* populations from strongly seasonal climates [62,63]. None of the above genes have yet been linked to circadian evolution, although *Prosap* does have previously documented signatures of local adaptation [64].

For the narrow signal at region B8, the highest PBE SNPs were observed at the upstream end of *retinal degeneration B* (*rdgB*; **Fig 7B**), involved in phototransduction, olfaction, and phospholipid transport [65–67]. Notably, the narrow PBE signal at region B11 (**Fig 7C**) centered on a gene that was recently detected as a top outlier in the 1975 to 2015 US temporal population genomic study cited above [14]; *Hexosaminidase 2* (*Hexo2*) encodes a sperm-associated protein that may play a role in fertilization [68,69]. Whereas another narrowly focused top outlier (region B5) centered on the noncoding RNA gene *CR43835* (**Fig 7D**), for which no function is known.

### Genomic and temporal distributions of outlier frequency changes

As hinted by the exclusively X-linked examples shown in **Fig 7**, for the 1933 to 2010s scan specifically, there was an abundance of X-linked loci among the top outliers: although the X chromosome constitutes less than one fifth of the analyzed genome, 12 of the top 15 outlier regions were X-linked (**S8 Table**). Explanations of this enrichment could include greater X chromosome effects of sampling variance (although this effect should have been stronger for the 1800s scan), genetic drift, or positive selection (potentially facilitated by X chromosome hemizygosity [70]). However, neither scan was meaningfully enriched for X-linked loci if the full list of outliers was considered (with the X contributing 21.7% of outliers for scan A and 18.6% for scan B). The excess of top outliers specifically for scan B could indicate that more of the X chromosome’s selection in this interval was from hard sweeps (as suggested by [71]) and therefore more readily detectable from window genetic differentiation [43]. However, the narrower peaks of differentiation shown in **Fig 7** could indicate a role of X-linked soft sweeps as well.

Among the top outliers discussed from each period, none appeared within the full list of 1% PBE outliers from the alternate time period. At least for genes promoting adaptation to local environmental conditions (such as temperature or day length), we might predict that alleles driving adaptation in the 1933 to 2010s time interval would have been adaptive in the 1800s to 1933 time interval as well. It is possible that each local population initially possessed only a subset of adaptive variants, and others arrived later via migration (or mutation). Alternatively, in the 1800s to 1933 interval, the frequencies of causative variants at some genes may have still been too early in their rise to generate extreme outlier PBE signals, but poising them to rise more quickly in the 1933 to 2010s interval. Another possibility is contingency—some variants may have only become beneficial after other adaptive changes had occurred due to epistatic interactions among circadian genes. Alternatively, this lack of outlier overlap could indicate that selective pressures differed between eras or that adaptive events tended to complete within a given time period rather than spanning between them.

### Further insights into previously implicated selection targets

Three other top outliers for the 1933 to 2010s interval included genes previously implicated in recent positive selection in European *D*. *melanogaster*. *Prosap* (in region B6) is thought to encode a structural component of the postsynaptic density [72] and affects circadian rhythm [73]. This gene was previously found to display signals of parallel adaptation across cold-adapted *D*. *melanogaster* populations [64], and it harbors a polymorphic transposon insertion associated with signals of selection [74]. Region B7 is centered on *phantom* (*phm*), which encodes a cytochrome P450 protein involved in ecdysteroid biosynthesis [75]. Variation at this gene revealed a selective sweep signal in a European *D*. *melanogaster* population [76], and the protein displays rapid evolution between species [77]. Further down the list, the region B21 contained *polyhomeotic-proximal* (*ph-p*), a developmental and chromatin-modifying gene (e.g., [78,79]), which also displayed evidence of a selective sweep in a European population [80], associated with altered thermosensitivity of gene expression [81]. For these outliers, our findings offer insights regarding the timing of positive selection.

### Potential shifts in membranes and metabolism

We also applied a Gene Ontology (GO) enrichment analysis to the broader sets of top 1% PBE outlier regions, using the permutation approach initially described in [82], separately to the outliers detected from each temporal scan. From the 1800s to 1933 scan, some of the most enriched categories are related to cell shape and morphogenesis, including actin-mediated contraction and membrane invagination (**S9 Table**). Enrichment was also observed for membrane (and vesicle) organization, compatible with the existence of population differences in membrane lipid composition [83] and this time interval coinciding with the species’ earliest known occupation of northern Europe [15]. Curiously, “response to DDT” was also enriched in the 1800s to 1933 interval, due to 3 cytochrome P450 outlier genes other than *Cyp6g1* in separate outlier regions, potentially reflecting pre-insecticide roles of insecticide-related genes (as with the *ChKov* locus described above). For the 1933 to 2010s interval, insecticide-related GO categories were not enriched among outliers. Instead, metabolic processes dominated the top results (including amide, fatty acid, lipid, and peptide metabolism), and categories related to cell division were enriched as well (**S9 Table**). As with membrane composition, metabolism is also known to vary significantly between *D*. *melanogaster* populations from contrasting thermal environments (e.g., [84,85]).

### Implications and future prospects

To our knowledge, our samples include the oldest pinned insects from which genome sequences have been reported (extending the temporal horizon of insect museomics from the late 1800s back to the early 1800s). Overall, 96% of our specimens yielded analyzable genomes, and 73% yielded genomes of relatively high quality. Although data quality remains an important concern, these results are encouraging for future museomic studies of small invertebrates.

To our knowledge, *D*. *melanogaster* is the smallest animal from which historical genomes have been obtained. Surprisingly, that small size may have even been an advantage. Male *D*. *melanogaster* are smaller than females (roughly 1 mg versus 1.8 mg wet weight [86]), and yet all 11 males in our study yielded high-quality genomes, whereas only half of the 14 females gave comparable outcomes. It is possible that less voluminous insects such as male *D*. *melanogaster* experience a faster rate of desiccation relative to decay, which should improve the quality and quantity of available DNA.

The genomes of these museum specimens have added to our knowledge of not only recent evolution in *D*. *melanogaster*, but also the specimens themselves. Beyond confirming the sex of each fly, we inferred from relatedness that the Swedish early 1800s flies, which had been linked to 3 different scientists and 2 different localities (but share the same type of pin typically used by Fallén), were actually from the same time and place. These results highlight important challenges when working with very old specimens, namely the lack of detailed collection data and potentially imperfect record keeping through time. Intriguingly, the unsequenced lectotype specimen (also labeled Småland) is, however, mounted with a distinctly different pin. The lectotype may accordingly be one of Ljungh’s flies, as described in Zetterstedt’s *Diptera Scandinaviae*. The exact collection date of the sequenced flies is unknown, although it may have occurred by 1810 (see Introduction). Given the apparent acquisition of one of these flies by Ljungh, his death in 1828 puts a latest possible bound on the collection date. These circumstances imply that the flies examined here are the oldest surviving specimens of *D*. *melanogaster*, predating those described by Meigen in 1830 [87].

The expanse of existing knowledge regarding the biology and genome of *D*. *melanogaster* has greatly enhanced the insights we have been able to draw from our museum fly genomes. In turn, our results have implications for the extensive *Drosophila* research community. We have identified various loci that represent likely targets of adaptive evolution within specific recent time intervals, and in some cases, these genes have been found to impact traits relevant to known selective pressures in the recent history of *D*. *melanogaster* (e.g., circadian regulation, viral and insecticide resistance). Hence, our study provides a wealth of candidates for functional evolution that can be investigated in subsequent laboratory studies, through leverage of the molecular, genetic, and transgenic tools available in this model system. Although museum specimens for this (or any) species are finite, further historical genomic studies in *D*. *melanogaster* have the potential to greatly inform the spatial and temporal dynamics of adaptive evolution and population history, broadening the range of insights that can be obtained from genomic variation in this population genetics model species.

## Methods

### Samples and DNA extraction

Historical specimens of *Drosophila melanogaster* were sampled from the entomological collections of Biological Museum (Lund, Sweden: MZLU), Swedish Museum of Natural History (Stockholm, Sweden: NRM), and National History Museum Denmark (Copenhagen, Denmark: NHMD) (**S1 Table**). DNA was extracted from the sampled specimens using the QIAamp DNA Micro kit (Qiagen). Given the fragility of these old samples, special attention was given to preserve their integrity. The samples were placed in a microtube without removing the pin. In cases where the pin was too long to fit in the microtube, or in the case the specimen was not mounted near the end, the pin was shortened. The lysis buffer was carefully added to avoid damaging the specimens due to surface tension of the buffer prior to the addition of the Proteinase K. The microtubes were incubated at 56°C for 2 h without shaking. The buffer containing the DNA was transferred to a new microtube, and 1 mL of MilliQ water was added to the tube containing the fly to wash away the buffer. After 30 min, the water was transferred to a new microtube and stored at −20°C for future DNA extraction if needed. Then, the fly was washed in successive ethanol solutions of increasing concentrations (1 mL, 50%, 70%, 80%, 99%), again paying extra attention to not harm the specimen. And finally, 500 μl of acetone was added to the specimen, which was then placed to dry in a suspended position. The rest of the DNA extraction followed the protocol, except for the final elution which was performed in 2 steps. In the first step, 50 μl of elution buffer was added to the columns, which were left to incubate at room temperature for 15 min prior to centrifugation. For the second step, 50 μl of elution buffer was added and the columns were incubated at 70°C for 15 min prior to centrifugation.

### Library prep and sequencing

The quality of the extracted DNA was visually investigated on an agarose gel (1.5%). The library prep followed mainly the protocol of [7]. Briefly, the fragmented DNA was blunt-end repaired with T4 Polynucleotide Kinase (New England Biolabs) and then purified with the MinElute Purification Kit (Qiagen). Next followed adaptor ligation, reaction purification, and adapter fill in. The resulting products were subsequently indexed with unique dual indexes. Indexing PCR was performed in 10 independent reactions to reduce amplification bias. Each PCR reaction consisted of 12 to 18 cycles depending on the concentration. Indexing PCR reactions were pooled prior to purification with Sera-Mag SpeedBeads carboxylate-modified hydrophilic (Sigma-Aldrich). An initial bead concentration of 0.5× was used to remove long fragments that were likely contaminants of fresh DNA. Libraries were selected using a 1.8× bead concentration to size select the intended library range of approximately 300 bp. The resulting libraries were quantified and quality checked using the Quanti-iT PicoGreen dsDNA assay and Bioanalyzer 2100 (Agilent Technologies). The final indexed libraries were pooled together prior to sequencing on an Illumina Novaseq 6000 platform at the Swedish National Genomics Institute (NGI) in Stockholm (2 × 150 bp, S4 flow cell). After observing that sequenced fragments only averaged about 50 bp in length, subsequent sequencing of most specimens with 2 × 50 bp reads was performed by the University of Wisconsin Biotechnology Center using an Illumina NovaSeq 6000 (**S1 Table**).

### Genome alignment and variant calling

The procedures for alignments and variant calling used to process the museum genomes largely followed the *Drosophila* Genome Nexus (DGN) pipeline as described in [88]. Using this pipeline with the release 5.57 *D*. *melanogaster* reference genome allowed newly assembled museum genomes to be compared against published data from modern population samples of individual genomes. Modern genomes from France and Egypt were reported in the subsequent DGN publication [22]. A sample from the Netherlands was also aligned in that study and originally sequenced by [89]. Population samples from Sweden (Stockholm) and Italy were sequenced by [51] and assembled using the DGN pipeline for this study. The (distinct) processing of published pooled sequencing (pool-seq) data from Lund, Sweden [90] is described in the next section.

Following read trimming using fastp (v0.21.0 [91]), we followed the DGN pipeline to map, filter, and call variants from the sequenced museum genomes. While the pipeline is described in detail in the previously cited papers, we note its key feature of 2 rounds of mapping prior to variant calling. The initial mapping of reads to the reference genome is performed with bwa -aln v.0.5.9 [92] and stampy [93]. Again following the DGN pipeline, this step is followed by indel and variant calling using GATK with default parameters [94,95]. We required heterozyous SNP calls to be supported by a minimum of 25% of the aligned reads for each called allele. The initially called SNPs and indels were applied to create an edited version of the (release 5) reference genome, to create a more robust target-specific reference for a second round of alignment of the reads with bwa -aln and stampy [88]. It was only after this second realignment and a shift of coordinates around indels to match the reference genome that variant calling for further downstream analyses was performed.

Diploid variant calling was initially performed at all sites, except for the X chromosomes from male flies. The sex of the flies in the museum samples was not identified prior to sequencing. It was identified by calculating the ratios of average read depth in the X chromosome versus the autosomes, which is expected to be approximately 1:1 in females and 1:2 in males. All perl scripts referenced in this section and elsewhere were executed on perl v5.34.0, all python scripts were executed on python 3.8.6 (except for the stampy aligner, which was run on python 2.7.18–3 to ensure compatibility), and the R scripts were run using R v.4.2.2.

For those museum genomes with a total coverage of <80% and average per-site read depth of <10, we concluded that diploid variant calls could not be confidently made. These were instead treated as “pseudo-haploid” with only a single nucleotide called per site; UnifiedGenotyper (GATK [94]) was run in haploid mode for all chromosomes for these low-quality genomes. When more than a single base was identified at a (pseudo-haploid) site, we retained the most frequent allele (or selected one at random in cases of a tie).

### Processing and analysis of pool-seq data

Published pool-seq data [90] was obtained from modern 3 Lund population samples: 1 from summer 2014 (SRR 5647735) and 2 from summer 2015 (SRR8439151 and 8439156). Each was derived from 40 wild-caught males. For each of these samples, alignment and post-mapping processing was performed using the same procedures as for individual genomes in the first round of the DGN pipeline, and the 3 pool-seq sample alignments were then merged to a single bam file. Because of the aggregate, multi-individual nature of these sequence data, frequency estimation was performed using PoolSNP [96]. We imposed an additional constraint of requiring there to be at least 5 instances of a minor allele for a site to be identified as polymorphic when filtering the VCFs, in order to minimize mislabeling read base-calling errors as SNPs. Additionally (and obviously), because individual genomes cannot be identified from pool-seq data and thus could not be used to create a revised reference genome, only a single round of alignments and variant calling was performed on the pool-seq data.

Pool-seq data introduces several statistical sampling artifacts that are not an issue with individual sequencing. Specifically, the number of reads contributed by each individual varies, so that some genomes are overrepresented and others are underrepresented. To deal with this, we calculate an effective allele (lineage) count [97], i.e., the number of *j* unique lineages at a site given *n*_*r*_ reads and n_c_ the number of chromosomes in the sample pool (*n*_*c*_ = 240 for autosomes, *n*_*c*_ = 120 for X chromosomes because all of the 120 flies sampled in the pool-seq study were male). The effective count *j* follows a distribution based on Stirling numbers of the second kind *S*(*n*_*r*_,*j*), i.e., the number of ways in which *n*_*r*_ reads can be partitioned into *j* lineages. The probability of *j* independent lineages in the sample is then:

$$P(j|,n_{c},n_{r})=S(n_{r},j)\frac{n_{c}!}{(n_{c}-j)!\;n_{c}^{n_{r}}}.$$

The effective number of distinct lineages (i.e., de facto allele count per site) was estimated from the expectation of this distribution. We found a maximum pool size of 183 for the autosomes (median = 145) and of 95 for ChrX (median = 70). Allele frequencies at each site were multiplied by the pool size to generate the appropriate downsampled counts for population genetic analysis.

### Assessment of genomic data quality

The total number of reads and the average read length was assessed from each bam file using CollectInsertSizeMetrics from the Picard suite of tools. Based on the depth and coverage criteria given above, the low-quality genomes that were treated as pseudo-haploid in the analysis pipeline were 18SL4, 18SL5, 18SL6, 18SL12 (from 1800s), 19SL3, and 19SL7 (from 1933). One sample from 1933, 19SL2, was of such low quality that it was excluded from any further processing or analysis.

To determine whether low-quality sequences contributed a significant number of false positive variants in each population sample, we tallied the number of “singletons”—instances where a given genome had an allele otherwise not observed among any other analyzed genome across populations. The fraction of singleton alleles that were A or T bases was calculated to determine if cytosine deamination accounted for many of the singletons, and whether this phenomenon was correlated with sample quality. Singleton minor alleles were excluded from certain downstream analyses, such as demographic inference, while a total allele count of at least 4 was required for all analyses unless otherwise indicated. We also calculated the Pearson correlations between read depth and coverage (as well as between read depth and the fraction of singletons contributed by each genome, and between the fraction of singletons and the A/T content) to quantify the association between these quality metrics.

### Identification of identity by descent

IBD among certain genomes in both the 1800s and 1933 Lund samples was initially implicated based on highly variable pairwise differences among genomes. We therefore set out to identify and mask redundantly sampled sequences in as targeted a manner as possible, while leaving maximal sample sizes of unrelated chromosomes at each locus. We implemented an IBD identification and masking framework similar to that employed by the *Drosophila* Genome Nexus [88] to mask specific regions of individual genomes that are affected by IBD. To identify specific regions of individual genomes with IBD, we began by partitioning chromosomes into “windows” defined by having 25,000 SNPs in an ancestral range Zambia population sample [88], averaging approximately 100 kb in length.

We then sought to account for genome-specific differences in genetic distances, such as due to sample quality. For each chromosome window of the *i*th genome, the background genetic distance *d*_*i*_ is the median Hamming distance between the *i*th genome and unrelated modern Lyon samples. This genome-specific individual distance estimate was used to obtain a distance correction factor *m*_*i*_ that accounts for its tendency to yield higher or lower distances than others from its population sample. The baseline population-specific distances *D* for 1800s and 1933 Lund were estimated as the median pairwise distance among individuals with no obvious IBD based on the Hamming distance matrix. Pairwise distances were compared to this baseline, scaled with respect to the Lyon outgroup distance, as follows.

For 2 genomes *i*,*j* collected in the same location and time point, the chromosome window Hamming distance is *d(i*,*j)*, while *m*_*i*_, *m*_*j*_ respectively are the scaling factors specific to sequences *i*,*j*—calculated from *d*_*i*_,*d*_*j*_ divided by the median pairwise distance between all of the Lund sequences (of a given time point) and the Lyon sequences.

- For the rescaled distance *D’(i*,*j) = d(i*,*j)m*_*i*_*m*_*j*_, a chromosome window is considered to be IBD between 2 diploid sequences (autosomes or female X chromosomes) if *D’(i*,*j) <* (7/8)*D*, i.e., closer to the predicted IBD distance of (3/4)*D* than to the outbred background distance *D*. The reasoning is that if 1 allele from each individual is IBD, then ¼ of pairwise allelic comparisons will yield zero distance due to this IBD, and hence, the overall expected distance is ¾ of that from a non-IBD pair. For haploid to haploid (male X chromosome comparison), the same rule with a factor of ½ is applied, while for haploid to diploid (male to female X chromosome comparison) a factor of ¾ is used.
- When pairwise IBD regions are identified, this window is masked in 1 genome of the pair. In a pairing of a low-quality and high-quality genomes, the masking is prioritized so that low-quality genomes are preferentially masked in order to minimize data loss.
- To avoid false negatives due to IBD regions covering partial windows, when a window was masked, the proximate half of the “upstream” and the “downstream” window was also masked. While this practice may mask some non-IBD regions, it errs on the conservative side for avoiding oversampling IBD alleles (since IBD covering more than half of a flanking window would be likely to satisfy the above masking criteria and cause the full window to be masked).

In addition to IBD among genomes, there was also evidence for depleted heterozygosity as a consequence of inbreeding in a subset of genomes from both the 1800s and (especially) the 1933 Lund populations, i.e., intragenomic IBD. In non-inbred genomes, the heterozygosity should approximately equal the population nucleotide diversity (*π*). Therefore, to identify enrichment of homozygosity in inbred genomes, we compared the observed heterozygosity per window to the median pairwise distance among non-IBD pairs from the same population in that window. If the within-window heterozygosity of a sample was less than *π*/2, the window was flagged as IBD and a randomly selected allele of the 2 in the window was masked.

We included samples believed to originate from other sites and time points (18DZ5 from Zealand, Denmark and 18SL6, which was labeled as being from Smäland, Sweden) in the IBD analyses, because the latter showed low divergence when compared to some of the 1800s Lund samples. There was unambiguous evidence of close relatedness between 18SL6 and 18SL10, suggesting that the former was in fact from Lund and thus could be grouped with this population in subsequent analyses. In contrast, 18DZ5 showed roughly 1% of its genome with apparent IBD with 1 Lund genome, and lesser levels with 2 others. As these could be false positives due to conservative filtering criteria, we continued to treat this sample as being from Zealand rather than Lund in demographic analyses based on location, while at the same time masking that small region of the genome (18SL12) that showed low, IBD-like divergence with respect to 18DZ5.

### Analysis of genetic differentiation

Temporal and spatial divergence among different populations (defined by time and location) was assessed with analyses of population genetic distances. Our analysis focused on X chromosome sequences (to the exclusion of low recombination regions defined by [64] because of the rarity of sex chromosome inversions in *Drosophila*, and only considered polymorphic sites without missing data in any of the analyzed samples).

To avoid distortions due to sample sizes, the number of alleles taken from each population (i.e., the 1933 Lund and the modern European and Egyptian samples) was downsampled to match the 4 alleles from the IBD-masked 1800s Lund allele counts. This entailed selecting 4 random independently sampled chromosomes at each site for the 1933 Lund population, the 2010s Lund pool-seq population, and the other modern populations, sampling independently at each analyzed site. This downsampled data was used to calculate nucleotide diversity within each population, as well as between-population genetic distance (*D*_*xy*_) and *F*_*ST*_ [98].

Genetic differentiation among the populations was also characterized using PCA. Downsampling of the Lund 1933 and modern samples was necessary to avoid over-weighting larger samples. However, because individual haplotypes were projected into the PCA space, the pool-seq Lund sample had to be excluded. To increase the number of sites with no missing data, we sampled non-masked X chromosomes that had no detectable pairwise IBD from the Hamming distance matrices from the 1800s and 1933 populations. Five such unrelated X chromosomes were identified from the 1800s samples: 18SL11, 18SL13, 18DZ5, 18GP9, and 18GP25, which were matched by the 5 unrelated 1933 Lund samples 19SL8, 19SL15, 19SL16, 19SL20, and 19SL23. Five X chromosomes were sampled from each of the modern European and Egyptian outgroup populations as well, for a total of 35 chromosomes (5 from 7 populations). For each of the 25,094 polymorphic sites with no missing data across all 35 samples in the analyzed regions of the X, the frequency of the minor allele at each site was calculated. PCA was then used to compute the 35 eigenvectors of the correlation matrix.

### Inversion polymorphism

To assay for inversions, we used the list of inversion-linked SNP alleles identified by [25,96] for the following common autosomal inversions: *In(2L)t*, *In(2R)Ns*, *In(3L)P*, *In(3R)K*, *In(3R)Mo*, and *In(3R)P*. The frequency of each inversion was estimated from the median frequency of the inversion-associated alleles among their respective loci. For the Lund pool-seq data, the requirement of more than 4 minor alleles per site to call minor alleles was relaxed to identify low-frequency inversions, i.e., even singleton minor alleles were included.

We used the median rather than the mean allele frequency because some alleles may be absent due to missing data. Additionally, not all of the alleles identified previously [25] as inversion-associated appeared to be fully linked to inversions in our data set. There were several instances where a small fraction of inversion-linked alleles are present in the museum samples or modern Lund (e.g., for *In(3R)P*, 4 of the 20 inversion-linked alleles were present at low frequency in the modern Lund pool-seq data), while the absence of inversion-linked alleles at the majority of sites suggested that the inversion itself is absent. In such cases, the use of mean allele frequency as a proxy for inversions would result in a nonzero estimated inversion frequency that is much lower than the reciprocal of the number of chromosomes sampled in the population. The estimated frequencies for each inversion in modern Lund versus the historical populations were compared with Fisher exact tests on karyotype counts, using R.

### Demographic estimation

Using allele frequency data from 1800s, 1933, and 2010s Lund, we estimated local effective population size (*N*_*e*_) for the 1809 to 1933 and 1933 to 2015 intervals by implementing a simple drift-only neutral model of evolution (thus assuming no effect of migration, selection, or other processes on allele frequency changes). To estimate *N*_*e*_ for these 2 time intervals, we compared the mean changes in allele frequencies across observed segregating sites to changes in allele frequencies simulated under a Fisher–Wright (FW) model of random genetic drift in discrete, non-overlapping generations. Specifically, we implemented a Bayesian approach to optimize *N*_*e*_ that minimizes the difference between observed $\bar{\Delta p}$, the mean absolute value difference in allele frequency across sites between 1809 and 1933 (and between 1933 and 2015), and the $\bar{\Delta p}$ simulated under an FW model of genetic drift.

We initialized the simulations by calculating the allele frequencies at polymorphic loci at each time point in the Lund populations. Regions of low recombination, as defined by [64] were excluded, as were singleton variants and non-Lund 1800s genomes. The sample frequencies *p*_*i*_ in the 1809 population (or 1933 for the second time interval) at the *i*th locus were used to approximate the initial allele frequencies *f*_*i*_ in the population using Bayesian estimation. The prior of *f*_*i*_ was based on a mutation-drift equilibrium with a site mutation rate of 5.21 × 10^−10^ per generation [99] and a candidate haploid effective population size N*. The posterior probabilities of *f*_*i*_ were calculated using Bayes’ Theorem from the prior probability distribution and the observed frequencies at each site. The initial allele frequencies in each simulation replicate were generated by drawing *n* alleles equal to the number in each sample. Using a uniform prior instead of mutation-drift equilibrium distributions resulted in very similar posterior distributions of *f*_*i*_.

Population allele frequencies were initialized to *f*_*i*_*(0)* at simulation start time, representing 1809 and 1933 for the first and second time intervals, respectively. The frequencies in generation t+1 were generated by drawing a binomial sample ~ *bin(f*_*i*_*(t)*, N*) at each site, where *f*_*i*_ = *x*_*i*_/*N* for *x*_*i*_ count of what was the minor allele at *t* = 0. The model assumes a sufficiently large gene pool for sampling with replacement and treating each locus as statistically independent (i.e., under linkage equilibrium). This sampling is repeated for 1860 (non-overlapping) generations for 1809 to 1933 and 1230 generations for 1933 to 2015, i.e., for 15 generations per year [16,17]. In the final (census) generation, an autosomal allele count *n* = 14 (*n* = 9 for ChrX) was drawn to represent the 1933 Lund sample, giving terminal sample allele frequencies *f*_i_*(T) based on *~ bin(f*_*i*_*(T)*, *n)*.

Much the same method was used for estimating the best fit *N*_*e*_ for the 1933 to 2015 time interval, with an important exception. Because the 2010s Lund samples contributed pool-seq data, the additional sample variance that is introduced by sampling of both individuals and reads must be incorporated into the simulation. In the terminal (census) generation, the simulations implemented a two-step sampling model consistent with the nature of pool-seq data. Namely, we sampled 240 alleles (corresponding to the 120 flies) in the census generation ~ *bin(f*_*i*_*(T)*, *240)*. This created a pool of 240 alleles where the frequency of the (initial) minor allele frequency was *f*_*i*_*’(T)*, with an expectation equal to *f*_*i*_*(T)*. To simulate the final sample set, we drew *n* alleles from this pool-seq subset of individuals (where *n* is the effective number of alleles per site sampled in the pool-seq data, with median values of 145 for autosomes and 70 for ChrX), i.e., *~ bin(f*_*i*_*’(T)*, *n)*. This final draw gave terminal sample allele frequencies *f*_i_*(T).

The absolute differences between final and initial sample allele frequencies in the simulations Δ*p_i_* = |*f*_i_*(T)–*p*_*i*_*(0)*| were averaged across polymorphic sites, giving $\bar{\Delta p}$. We optimized our estimate of *N*_*e*_ by running the FW simulations over 1,000 replicates over a range of N* = {1,000…20,000} in increments of 1,000 and selecting the value minimizing the difference between simulated and observed $\bar{\Delta p}$. This estimate was further refined by considering N* in 5 increments of 100 on either side of the coarse-grained optimum.

We evaluated the accuracy our estimates of effective population size (*N*_*1*_, *N*_*2*_) for the time intervals 1809 to 1933 and 1933 to 2015 by comparing *π* observed in the 1800s, 1933, and 2010s Lund samples to that of simulated populations of sizes *N*_*1*_, *N*_*2*_ at these time points. The simulations were initiated by sampling *N*_*1*_ alleles from a source European population based on the inferred demographic history of *D*. *melanogaster* [30]. The size *N*_*1*_ Lund sample then experienced genetic drift (with no gene flow or natural selection) for 1860 generations (scaled in units of 4*N*_*e*_) until 1933, at which point the population size was set to *N*_*2*_ and the simulations continued for a further 1230 generations. This neutral demographic model was implemented using the program ms [100], with the mutation rate cited above, along with a crossing-over rate of 1.09 × 10^−8^, a gene conversion rate of 6.25 × 10^−8^, and mean gene conversion tract length of 518 bp [101]. We focused ms simulations on chromosome arm 3L because of the favorable sample sizes, the relative rarity of inversion polymorphisms on this arm, and the availability of a parameterized demographic model for its evolutionary history in Europe.

### Population branch excess scan for positive selection

To identify regions of the genome that may contain sites that may have experienced directional selection in the time between 1800s, 1900s, and modern samples, we used PBE [31], a statistic that aims to identify loci with particularly strong allele frequency differentiation in a focal population, in light of data from 2 outgroup populations. PBE is based on the PBS [32], which essentially estimates the length of the focal population’s branch (in terms of allele frequency differentiation). PBE is determined by a comparison of the focal population’s observed PBS value to that expected based on the lengths of the non-focal populations’ branches at the same locus, along with the average lengths of the focal and non-focal branches across loci [31]. Compared to PBS, PBE is more focused on population-specific differentiation and will not return a large value at loci where all population branches are long (e.g., due to background selection or positive selection in all populations).

These statistics are based on log-transformed window *F*_*ST*_ values *d* = -log(1-*F*_*ST*_) between each pair of populations. Here as in other studies, window *F*_*ST*_ was calculated by summing the numerator terms and summing the denominator terms of the *F*_*ST*_ estimator [98], and obtaining their ratio. Chromosome windows were defined to contain 250 Zambia non-singleton SNPs, corresponding to approximately 4.6 kb windows on average. We performed a separate scan for each time interval. To focus on the 1800s, we made the 1800s genomes the focal population and used Lund 1933 and Lund 2010s as the non-focal populations. To focus on the 1900s, we made Lund 1933 the focal population and used Lund 2010s and Lyon (2010) as the outgroups. Although the Lund 2010s sample comes from (distinctly-processed) pool-seq data, the presence of one other individually sequenced population sample among the non-focal populations should prevent any artefactual differences between individual and pool-seq data from registering as differentiation specific to the focal population.

Given that a viable demographic model was not available to provide a distribution of PBE values under a null hypothesis, outlier PBE values were defined based on the upper 1% quantile of the window distribution (which contained 240 windows genome wide). Because strong selection can create linkage disequilibrium that extends across multiple windows, we aggregated adjacent or near-adjacent PBE-significant windows (separated by no more than 2 non-outlier windows). Although SNP-level genetic differentiation can sometimes allow the detection of soft sweeps that do not register as outlier in window-level scans, our sample sizes of museum genomes were not sufficient to enable this approach [43]. Instead, we used site PBE as a heuristic for identifying sites and genes of interest within window-based PBE outlier regions.

### Gene ontology enrichment

We performed a GO enrichment analysis on genes located PBE outlier regions from the 1800s- and 1900s-focused genome scans in order to establish whether certain functional categories (e.g., key metabolic or developmental pathways) were disproportionately overrepresented among genes that may differ between the historical and modern populations. The GO analysis methods followed the approach of [82], which involves randomly permuting the genomic locations of outlier regions, to account for variation in gene length and the genomic clustering of functionally related genes. Raw *p*-values were computed from 10,000 random permutations, based on the proportion of replicates with an equal or greater number of outlier regions associated with a given GO category as observed in the empirical data.

## Supporting information

S1 Table Sample information and genomic sequencing statistics. Available collection information is given for each museum specimen. All early 1800s Sweden flies (including the 1 labeled Småland) were ultimately inferred to have come from the same collection event. Sequencing statistics including read count and lengths, depth of coverage, genomic coverage, singleton rate, and proportion of singletons with A or T are also given. Sample 19SL2 was excluded from most analyses because of its low sequencing depth and genomic coverage. The sex of each specimen was inferred from the relative levels of sequencing depth on the X chromosome and the autosomes. Genomic characteristics of a representative set of genomes from modern material (from Lyon, France—Lack and colleagues (2016)) are provided, as depicted in Fig 1.
(XLSX)

S2 Table Evidence of identity-by-descent from relatedness and inbreeding and genomic masking proportions. **Tab A.** Mean pairwise genetic distance between X chromosomes from museum and modern genomes (excluding sites with missing data from any individual). Unusually low values are highlighted. Mean distance against a panel of modern genomes is indicated at the bottom. No genomes were found to have anomolously high distances to all others, suggesting that they all reflect *D*. *melanogaster* specimens. **Tab B.** Proportion of each genome masked due to pairwise IBD with other genomes, given as a fraction of the total genome and each chromosome arm. **Tab C.** Proportion of each genome masked due to inbreeding IBD, as indicated by runs of minimal heterozygosity, for each full genome and each chromosome arm.
(XLSX)

S3 Table Regions of each museum fly genome that were masked due to relatedness IBD. Release 5 positions are given in separate tabs for each chromosome arm.
(XLSX)

S4 Table Regions of each museum fly genome that were masked due to inbreeding IBD. Release 5 positions are given in separate tabs for each chromosome arm.
(XLSX)

S5 Table Results of principle components analysis of genetic structure. Loadings for each genome are given for all 35 principle components. The X chromosome was analyzed to avoid the influence of autosomal inversions. Up to 5 apparently unrelated individuals were included per population sample.
(XLSX)

S6 Table The estimated frequency of each of the most common inversions in museum and modern populations. Inversion frequency estimates and results from each SNP reported to be associated with each inversion (by Kapun and colleagues (2016)) are given in separate tabs. Estimation of inversion frequency was based on the median of SNP frequencies, in order to avoid spurious nonzero estimates.
(XLSX)

S7 Table Effective population size estimates from drift-only models do not predict observed nucleotide diversity. **Top:** Mean frequency differences and haploid effective population size estimates from drift-only models for each Lund time interval. **Bottom:** Comparison of nucleotide diversity from simulations (using estimated Ne) versus observed data, for arm 3L.
(XLSX)

S8 Table Outlier regions from PBE reflecting windows with SNP frequencies at strongly differing frequencies between samples. **Tab A.** For the 1800s time interval (1800s as focal population, Lund 1933 and Lund 2010s), PBE outlier regions based on the upper 1% quantile and overlapping genes are given. **Tab B.** For the 1900s time interval (Lund 1933 as focal population, Lund 2010s and Lyon as outgroups), PBE outlier regions based on the upper 1% quantile and overlapping genes are given. **Tab C.** For the 1800s time interval, PBS and PBE for all genomic windows are given. In addition to window values, the maximum SNP PBE and PBS are given. Nucleotide diversity, Dxy, and FST values are provided as well. **Tab D.** For the 1900s time interval, PBS and PBE for all genomic windows are given. In addition to window values, the maximum SNP PBE and PBS are given. Nucleotide diversity, *D*_*xy*_, and *F*_*ST*_ values are provided as well.
(XLSX)

S9 Table Gene Ontology (GO) enrichment based on PBE outlier regions. **Tab A.** Gene ontology enrichment for the 1800s-focused PBE outliers. **Tab B.** Gene ontology enrichment for the 1900s-focused PBE outliers. For each GO category, the total number of genes in the analyzed regions is given, along with the number of outlier regions associated with these genes, and the identities of these outlier-overlapping genes. A permutation *P*-value is also given for each GO category, which does not attempt to correct for the number of GO categories tested.
(XLSX)

## Abbreviations

DmelSV: *D*. *melanogaster* sigmavirus
FW: Fisher–Wright
GO: Gene Ontology
hDNA: historical DNA
IBD: identity by descent
NGI: National Genomics Institute
NHMD: National History Museum Denmark
PBE: population branch excess
PBS: population branch statistic
PCA: principal components analysis
